# Electrochemical DNA Cleavage Sensing for EcoRV Activity and Inhibition with an ERGO Electrode

**DOI:** 10.3390/bios14020073

**Published:** 2024-01-30

**Authors:** Da Eun Oh, Hyun Beom Kim, Tae Hyun Kim

**Affiliations:** Department of Chemistry, Soonchunhyang University, Asan 31538, Republic of Korea; dani0607@sch.ac.kr (D.E.O.); kimhb93@sch.ac.kr (H.B.K.)

**Keywords:** endonuclease, EcoRV, electrochemical assay, graphene

## Abstract

An electrochemically reduced graphene oxide (ERGO) electrode-based electrochemical assay was developed for rapid, sensitive, and straightforward analysis of both activity and inhibition of the endonuclease EcoRV. The procedure uses a DNA substrate designed for EcoRV, featuring a double-stranded DNA (dsDNA) region labeled with methylene blue (MB) and a single-stranded DNA (ssDNA) region immobilized on the ERGO surface. The ERGO electrode, immobilized with the DNA substrate, was subsequently exposed to a sample containing EcoRV. Upon enzymatic hydrolysis, the cleaved dsDNA fragments were detached from the ERGO surface, leading to a decrease in the MB concentration near the electrode. This diminished the electron transfer efficiency for MB reduction, resulting in a decreased reduction current. This assay demonstrates excellent specificity and high sensitivity, with a limit of detection (LOD) of 9.5 × 10^−3^ U mL^−1^. Importantly, it can also measure EcoRV activity in the presence of aurintricarboxylic acid, a known inhibitor, highlighting its potential for drug discovery and clinical diagnostic applications.

## 1. Introduction

Restriction endonucleases, also known as restriction enzymes, are proteins that recognize and cleave DNA at specific sequences called restriction sites [1,2,3,4]. They are part of the restriction-modification systems that protect prokaryotic cells from foreign DNA, such as viruses. Restriction endonucleases belong to the endonuclease family of enzymes, which are involved in various biological processes. Due to their ability to cut DNA precisely, restriction endonucleases have been widely used in genetic engineering and biotechnology, and as potential candidates for antimicrobial and antiviral therapies. EcoRV, a restriction enzyme derived from Escherichia coli, stands out as one of the extensively studied endonucleases [5,6,7,8,9,10]. Functioning as a type II restriction endonuclease, EcoRV exhibits the ability to cleave duplex DNA precisely at the TA site within the target sequence GATATC, thereby producing blunt ends [11,12]. The overexpression of EcoRV prompts heightened activation of DNA repair mechanisms in cells experiencing DNA damage, representing a notable bacterial defense mechanism against drug treatments. This overexpression of EcoRV enzymes serves as a bacterial strategy to counteract the effects of antibacterial agents by robustly repairing damaged DNA [13]. Numerous research groups are actively exploring potential candidates with nuclease inhibitory properties. These candidates hold promise in preventing DNA repair by nucleases, offering a potential avenue for the treatment of bacterial diseases [14,15]. Traditional methods for assessing enzyme activity, such as high-performance liquid chromatography (HPLC) [16,17], gel electrophoresis [18], and immunoaffinity [19], have been widely used in enzyme analysis. However, these conventional assays are often time-consuming, labor-intensive, discontinuous, and expensive. Seeking to overcome these limitations, researchers have turned to alternative approaches like fluorescent, electric, and colorimetric assays utilizing labeled substrates or reagents, enabling simpler, continuous, and real-time measurements of endonuclease activity [20,21,22]. Despite the advantages of these novel methods, they still depend on bulky and costly instruments, limiting their suitability for the development of cost-effective and portable monitoring devices. Electrochemical methods present a compelling alternative with numerous benefits in biomolecule determination and quantification, offering simplicity and cost-effectiveness [23]. Additionally, electrochemical methods are more amenable to miniaturization compared to optical methods while maintaining comparable sensitivity.

**Scheme 1:**
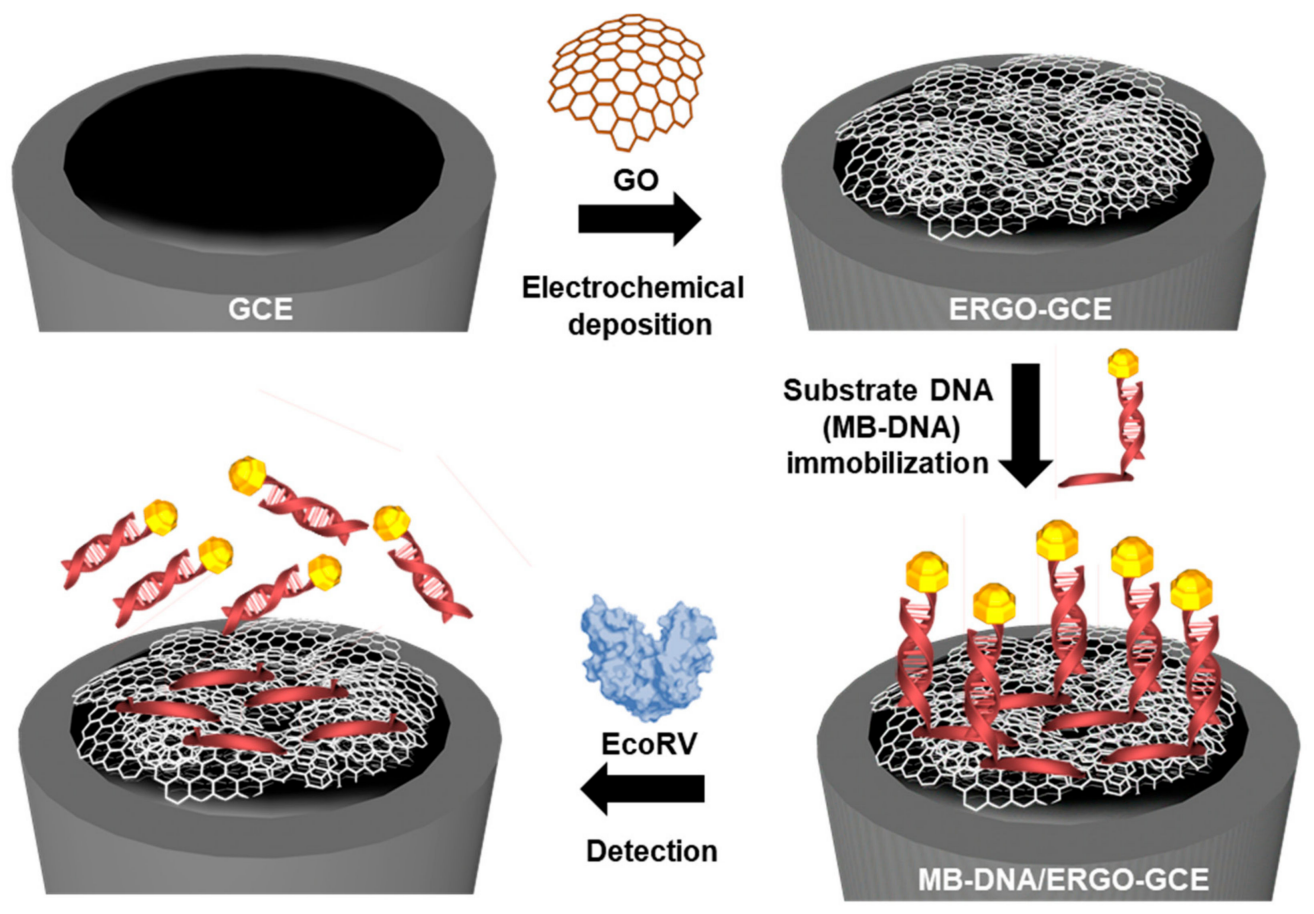
Fabrication of the substrate DNA-modified ERGO-GCE sensor for the detection of EcoRV activity and inhibition.

In this study, we present an electrochemical method for measuring the activity and inhibition of endonuclease EcoRV using an electrode of electrochemically reduced graphene oxide (ERGO) with immobilized substrate DNA (MB-DNA). The ERGO electrode markedly enhances sensor detectability and sensitivity, which is attributable to its conductivity, catalytic activity, and biocompatibility, along with a substantial surface area and ample reaction sites [24]. The MB-DNA complex consists of a single-stranded DNA (ssDNA) region attached to the ERGO surface via strong π-π interactions, a double-stranded DNA (dsDNA) region labeled with a redox indicator, and methylene blue (MB), for signal generation [25,26,27]. Scheme 1 illustrates the fabrication and sensing principles of the proposed electrochemical assay. Upon exposure to a sample containing EcoRV, the MB-DNA-immobilized ERGO electrode undergoes enzymatic hydrolysis. This process causes the cleaved dsDNA fragments to detach from the ERGO surface, subsequently lowering the concentration of MB near the electrode. Consequently, the electron transfer efficiency for MB reduction decreases, leading to a decrease in the reduction current. Achieving a direct, sensitive, and rapid quantification of EcoRV activity and inhibition is possible by measuring the cathodic peak current of the MB redox tag. This method offers advantages such as high sensitivity, simple operation, and adaptable design by modifying the substrate DNA sequence. These features make this method a promising tool for developing novel assays for drug screening and enzyme-related basic research.

## 2. Materials and Methods

### 2.1. Materials

The DNA substrates for EcoRV were purchased from BIONEER Corporation (Daejeon, Republic of Korea), and the sequences were 5′-Methylene Blue- AGT ATG ATA TCC A-3′ for upper strand DNA and 5′-CTAGCTATGTGC CGAATTTCAAGGACAGTT GTATGGATATCATAC T-3′ for bottom-strand DNA [22,28]. Graphite powder, potassium permanganate (KMnO_4_), hydrogen peroxide (H_2_O_2_), sodium nitrate (NaNO_3_), sulfuric acid (H_2_SO_4_), phosphate-buffered saline (PBS, pH 7.4, 10 mM), potassium ferrocyanide (K_3_Fe(CN)_6_), tris-buffered saline (TBS; 10 mM Tris-HCl), and aurintricarboxylic acid (ATA) were all purchased from Sigma-Aldrich, Burlington, MA, USA, and used without any treatment. We purified deionized water (DI water, 18 MΩ) using a MilliQ system (Millipore Korea, Co., Ltd., Seoul, Republic of Korea).

### 2.2. Instrumentation

CHI 660D (CHInstruments, Inc., Austin, TX, USA, Z-202306208148 at the Research Support Center for Bio-Big data Analysis and Utilization of Biological Resources) was used as an electrochemical workstation with a glassy carbon electrode (GCE, BAS MF-2012, 3 mm diameter) as the working electrode, a platinum wire (BSA MW-1032) as the counter electrode, and a silver/silver chloride electrode (Ag/AgCl, BSA MF-2052, Re-5B) as the reference electrode. Sonication was carried out with a VC505 Vibra-cell sonicator (500 W, solid Ti-Al-V tip; Sonics & Materials, Z-202308038894 at the Research Support Center for Bio-Big data Analysis and Utilization of Biological Resources). Raman spectra were recorded using an EnSpectr R532 Raman spectrometer (Enhanced Spectrometry, Inc., Torrance, CA, USA, Z-202312061405 at the Research Support Center for Bio-Big data Analysis and Utilization of Biological Resources) with a 532 nm laser excitation and a 30 mW laser power. Contact angles were measured using a contact angle analyzer (Phoenix mini, SEO Co., Ltd., Suwon, Republic of Korea, Z-202312061406 at the Research Support Center for Bio–Big data Analysis and Utilization of Biological Resources).

### 2.3. Fabrication of the Substrate DNA-Modified ERGO-GCE Sensor

GO was synthesized using a modified Hummers method [29]. The resulting GO was then dispersed in 10 mM PBS buffer (pH 7.4) and sonicated for 1 h to obtain a homogeneous yellow-brown solution (0.3 mg mL^−^¹). Before fabrication of the ERGO-modified glassy carbon electrode (ERGO-GCE), the GCE was carefully polished with progressively finer alumina powder slurries (1.0, 0.3, and 0.05 μm), sonicated for 10 min, and thoroughly rinsed with DI water. To prepare the ERGO-GCE, cyclic voltammetry (CV) was performed at the GCE in the GO solution (0.3 mg mL^−^¹ in 10 mM PBS buffer, pH 7.4) within a potential range of 0.8 V to −1.5 V vs. Ag/AgCl reference electrode, using a scan rate of 10 mV s^−^¹ for 3 cycles [30,31]. A duplex DNA substrate (MB-DNA) for EcoRV was prepared by mixing 20 µM MB-tagged short upper strand DNA with 20 µM of the long bottom-strand DNA in a pH 7.4 buffer containing 10 mM Tris-HCl and 10 mM NaCl. The mixture was then annealed by heating at 95 °C for 5 min and slowly cooled to room temperature over a 1 h period. The ERGO-GCE was subsequently immersed in a solution containing 1.5 µM duplex DNA substrate in 10 mM Tris buffer (pH 7.4) for 40 min. During this process, the duplexes formed strong bonds with the ERGO-GCE through π-π stacking interactions between the surface of ERGO and the single-strand region of the duplex DNA.

## 3. Results and Discussion

### 3.1. Characterization

**Figure 1:**
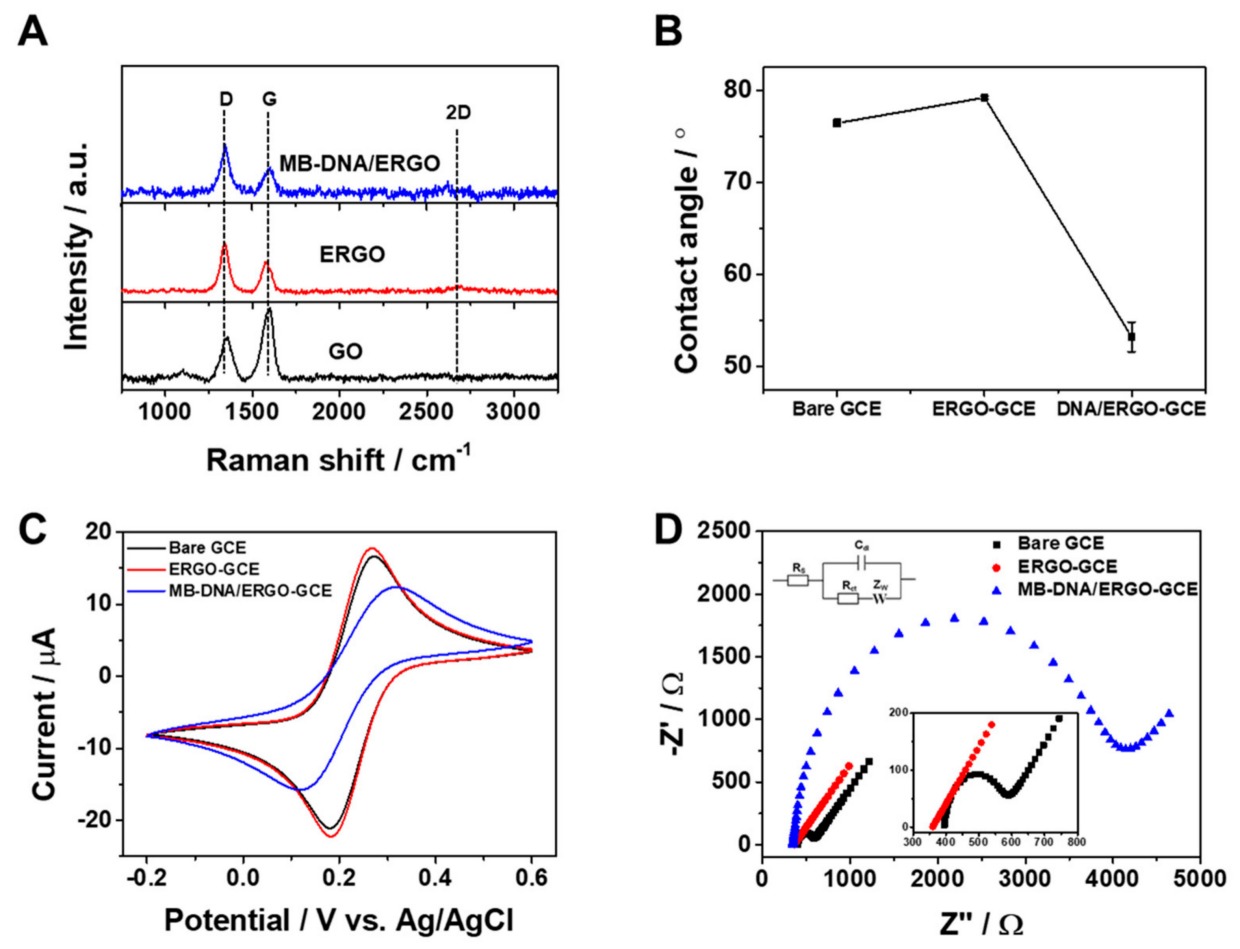
(**A**) Raman spectra of MB-DNA/ERGO, ERGO, and GO. (**B**) Contact angle measurements of water droplets on bare GCE, ERGO-GCE, and MB-DNA/ERGO-GCE. (**C**) CV curves recorded at a scan rate of 10 mV s^−1^ And (**D**) Nyquist plots of bare GCE, ERGO-GCE, and MB-DNA/ERGO-GCE in 10 mM Tris buffer (pH 7.4) containing 5 mM [Fe(CN)_6_]^3−^ inset: a Randles equivalent circuit diagram.

The successful formation of the ERGO-GCE, prepared by the electrochemical method, was confirmed by Raman spectroscopy. Figure 1A displays a typical Raman spectrum of MB-DNA and ERGO on the GCE, exhibiting the characteristic D, G, and 2D bands. These bands correspond to the intervalley scattering of disordered structures (D band), the first-order scattering of the E₂g mode for sp^2^-hybridized carbon (G band), and a second-order overtone of the D band (2D band), respectively [32]. Electrochemical deposition of ERGO on the GCE resulted in a characteristic shift of the D band in the Raman spectrum from 1358 cm^−^¹ to 1340 cm⁻¹ and the emergence of a 2D band at 2678 cm^−^¹, confirming the successful reduction of GO to ERGO. Furthermore, the intensity ratio of the D and G bands (I_D_/I_G_) increased from 0.57 to 1.64 during the reduction process, implying a decrease in the average size of the sp^2^ carbon domains by the elimination of oxygen-containing functional groups [33]. Additionally, for MB-DNA/ERGO, distinct D and G bands associated with ERGO are evident, alongside notable signals linked to MB at 591.9, 662.9, 769.5, 883.5, 1033.7, 1148.3, 1298.1, 1386.4, 1428.6, 1498.3, and 1622.1 cm^−1^ [34,35,36]. However, the overlap between specific Raman peaks of ERGO and MB, particularly at 1428.6 and 1622.1 cm^−1^, introduces challenges in conducting I_D_/I_G_ calculations. Nevertheless, it is affirmed that we can identify the presence of MB and ERGO individually. To confirm the deoxygenation of ERGO, we investigated changes in its wettability through contact angle measurements. Deoxygenation during GO reduction was expected to increase the surface hydrophobicity. Figure 1B and Table S2 present the wetting properties of GCE, ERGO-GCE, and MB-DNA/ERGO-GCE. The average equilibrium static contact angles for GCE and ERGO-GCE were 76° and 79°, respectively. While the GCE surface shows slight hydrophobicity, likely due to polishing or manufacturing processes, the electrochemical reduction of hydrophilic GO to ERGO successfully removed oxygen functional groups, resulting in a slightly more hydrophobic ERGO-GCE surface. Interestingly, the modification of ERGO-GCE with MB-DNA resulted in a drastic decrease in the contact angle from 79° to 53°. This significant difference is due to the negative charge of the phosphate groups in MB-DNA, which makes the surface more hydrophilic [37]. This finding confirms the successful formation of MB-DNA/ERGO-GCE. Each step of the modified electrode fabrication was also confirmed by examining the electron transfer kinetics of GCE, ERGO-GCE, and MB-DNA/ERGO-GCE using CV and electrochemical impedance spectroscopy (EIS) with [Fe(CN)_6_]³^−^ as a redox probe. Figure 1C presents their voltammetric responses, showing well-defined redox peaks. ERGO-GCE exhibited superior electrochemical performance with a smaller peak-to-peak separation (ΔE_p_) and enhanced peak currents (I_p_) compared to bare GCE, owing to the large surface area and electrocatalytic properties of ERGO. However, MB-DNA/ERGO-GCE exhibited an increase in ΔE_p_ and a decrease in I_p_, indicating hindered electron transfer due to repulsion between the negatively charged phosphate backbone of MB-DNA on ERGO-GCE and anionic [Fe(CN)_6_]^3−^. These observations confirm the successful fabrication of MB-DNA/ERGO-GCE. EIS analysis further validated the sensor fabrication by examining the charge transfer resistances (R_ct_) of the electrodes in Nyquist plots (Figure 1D). ERGO-GCE exhibited a smaller R_ct_ than GCE, indicative of faster electron transfer. Conversely, modifying ERGO-GCE with MB-DNA resulted in an increased R_ct_, confirming the hindered electron transfer and corroborating the formation of MB-DNA/ERGO-GCE biosensors.

**Figure 2:**
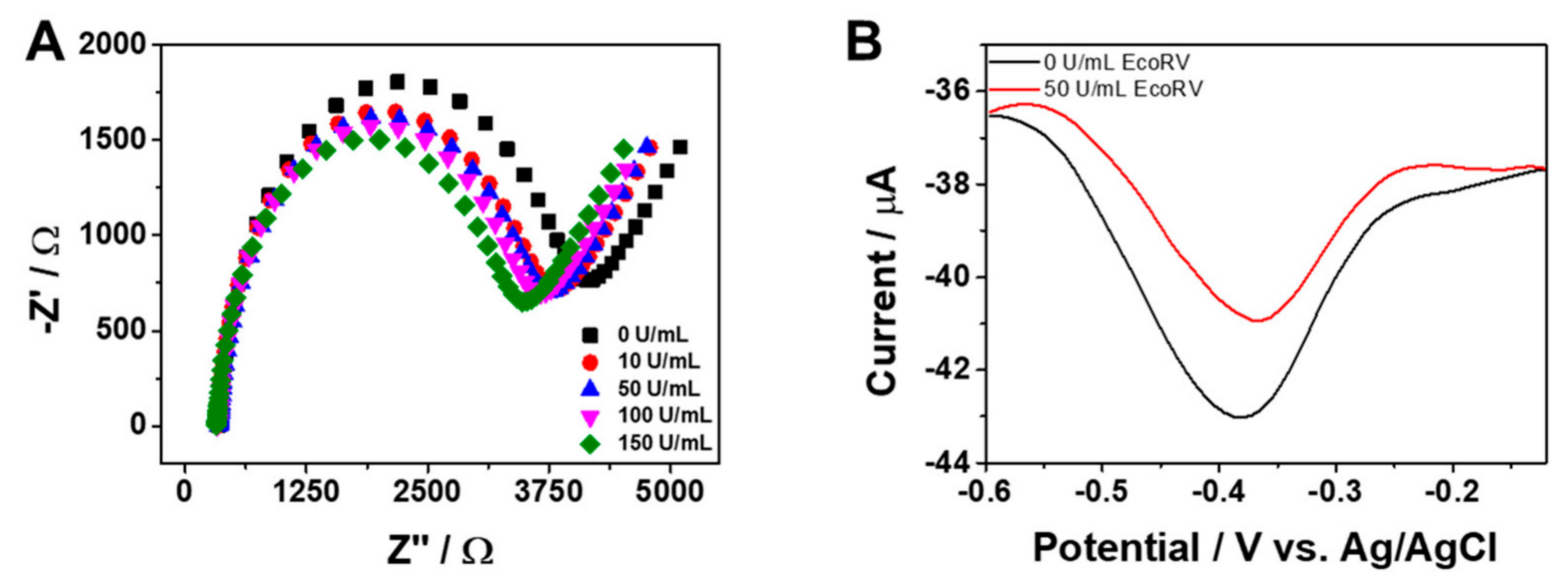
(**A**) Nyquist plots of MB-DNA/ERGO-GCE immersed in different concentrations of EcoRV (0, 10, 50, 100, and 150 U/mL) in 10 mM Tris buffer (pH 7.4) containing 5 mM [Fe(CN)_6_]^3−^. (**B**) DPV curves of MB-DNA/ERGO-GCE in the absence and presence of 50 U/mL in 10 mM Tris buffer at pH 7.4. The accumulation time for MB-DNA (1.5 µM) coating was 40 min and the incubation time for EcoRV detection was 5 min.

To validate the proposed sensing principle, we used EIS to measure the response of the MB-DNA/ERGO-GCE biosensor to different EcoRV concentrations (Figure 2A). As the EcoRV concentration increased, the charge transfer resistance (R_ct_) progressively decreased. This decrease is attributed to the enzymatic hydrolysis of MB-DNA, releasing the cleaved dsDNA fragment and decreasing the steric hindrance to electron transfer at the electrode surface. This confirms the feasibility of using the MB-DNA/ERGO-GCE biosensor for EcoRV activity detection. Given that MB-DNA incorporates an electroactive MB tag as an electrochemical indicator, the MB-DNA/ERGO-GCE can exhibit voltammetric sensing features. Assuming the addition of EcoRV leads to the cleavage of MB-DNA, resulting in a change in the voltammetric signal, the voltammetric sensing behavior was investigated using differential pulse voltammetry (DPV), a highly sensitive and fast method for low concentrations of a redox probe and irreversible redox reactions compared to other voltammetric techniques. Figure 2B illustrates typical DPV curves of the MB tag on the signal probe, with a reduction peak observed at −0.37 V. Upon the addition of 50 U/mL EcoRV, the MB-DNA/ERGO-GCE exhibited a decrease in I_p_ due to the slow charge transfer resulting from the release of the cleaved MB-tagged dsDNA region from the electrode, indicating the potential for voltammetric analysis using this sensor.

### 3.2. Optimization

**Figure 3:**
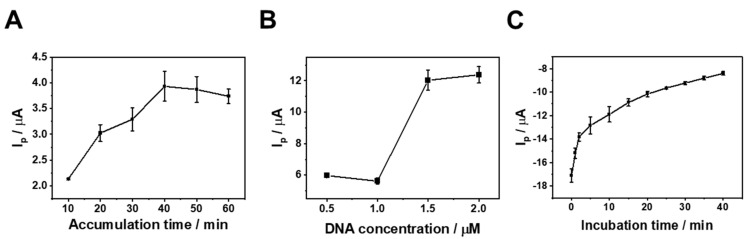
Effect of (**A**) MB-DNA accumulation time for immobilization, (**B**) MB-DNA concentration and (**C**) the incubation time on I_p_ values for EcoRV reaction on the MB-DNA/ERGO-GCE in 10 mM Tris-HCl buffer (pH 7.4). The current was measured in triplicate (*n* = 3).

To optimize the sensing performance, we investigated the influence of three key parameters: MB-DNA immobilization time and concentration and EcoRV incubation time (Figure 3). Figure 3A reveals that the voltammetric response increased steadily with the increase in the MB-DNA accumulation time until reaching saturation at 40 min. This indicates maximum MB-DNA adsorption on the ERGO-GCE surface, making 40 min the optimal accumulation time for sensor preparation. Figure 3B shows that a 1.5 µM MB-DNA concentration yielded the best analytical performance. Finally, Figure 3C demonstrates that the voltammetric response progressively increased with EcoRV incubation time, reaching a plateau at 5 min. Therefore, 5 min was sufficient for complete reaction, setting the optimal incubation time for subsequent applications. Based on these results, we optimized the working conditions of electrochemical sensing for the next application.

### 3.3. Electrochemical Sensing Performance

**Figure 4:**
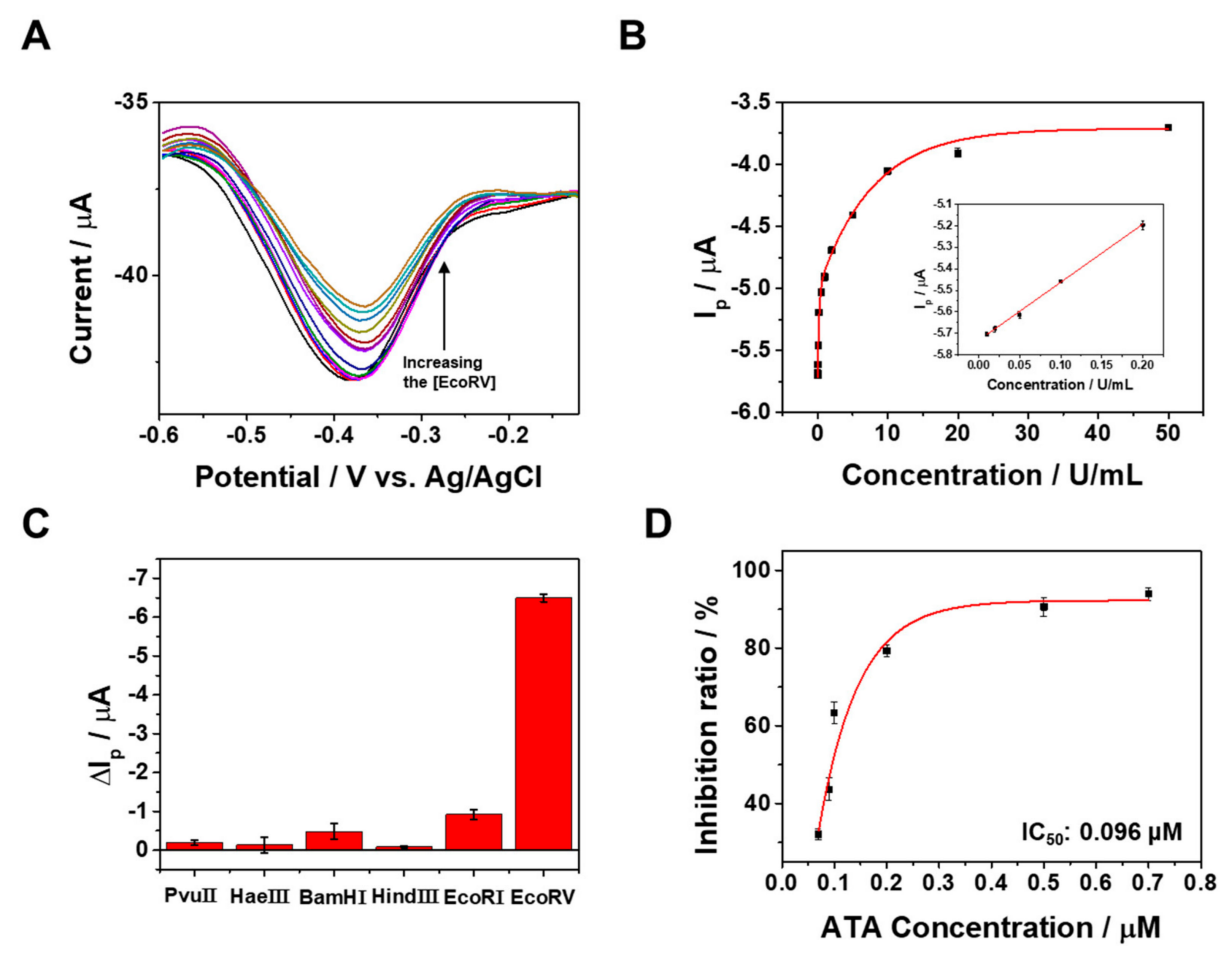
(**A**) DPV curves at MB-DNA/ERGO-GCE corresponding to the analysis of EcoRV in the range of concentration of 0.01 to 50 U/mL in 10 mM Tris buffer (pH 7.4). (**B**) The calibration curve corresponding to the DPV peak currents as a function of EcoRV concentration. (**C**) Selectivity of the proposed sensor over other competing nucleases. The concentration of EcoRV was 1 U/mL and the concentrations of the others were 20 U/mL. (**D**) EcoRV inhibition activity of ATA, determined in a dose-dependent manner. The current was measured in triplicate (*n* = 3).

Under optimal conditions, we evaluated the assay performance of the proposed method. In Figure 4A, the DPV curves display variations in response to different concentrations of EcoRV. The peak current at −0.37 V increases with the rise in EcoRV concentration from 0 to 50 U/mL (Figure 4B). Notably, a robust linear relationship between I_p_ and the EcoRV concentration is established in the range from 1.0 × 10^−2^ to 2.0 × 10^−1^ U/mL (inset of Figure 4B), described by the equation I_p_ (µA) = −5.7 + 2.7C (R^2^ = 0.9983), where C represents the EcoRV concentration (U/mL). Based on the standard deviation of the blank signal, the calculated limit of detection (LOD) is 9.5 × 10⁻^3^ U/mL. The LOD was calculated using the equation of 3 σ/s (σ: standard deviation, s: slope of the linear range). The electrochemical signals were measured in triplicate (*n* = 3) to obtain average value and standard deviations. The sensitivity of this method surpasses that of previous approaches for EcoRV detection (Table 1). The heightened sensitivity is primarily attributed to (1) the high selectivity of the EcoRV-induced cleavage reaction, (2) the efficient electron transfer facilitated by MB-DNA immobilization on conductive ERGO-GCE, and (3) the efficient detachment of the cleaved MB-tagged region from the ERGO-GCE. To assess the selectivity of our method, we employed a panel of five non-specific nucleases as negative controls: PvuII, HaeIII, BamHI, HindIII, and EcoRI. Each enzyme recognizes and cleaves a distinct DNA sequence, none of which overlap with the EcoRV recognition site present in the MB-DNA. Therefore, none of these controls were expected to trigger the cleavage of MB-DNA and subsequent release of the MB-tagged dsDNA fragment. As anticipated, negligible changes in the voltammetric responses were observed upon incubation with any of the negative control nucleases (Figure 4C). This confirms their lack of interaction with the MB-DNA. Conversely, a significant current decrease was evident upon EcoRV addition, underscoring its specific recognition and cleavage of the target sequence. This observation clearly demonstrates the ability of our method to distinguish EcoRV from non-specific nucleases, highlighting its high selectivity. To further showcase the capabilities of our MB-DNA/ERGO-GCE platform, we evaluated its potential for studying EcoRV inhibition activity. We used the inhibitor ATA, which is known to target EcoRV’s catalytic activity. As Figure 4D demonstrates, increasing the concentration of ATA led to a progressive rise in the inhibition ratio. This confirms that the presence of ATA effectively interferes with EcoRV’s ability to cleave the MB-DNA, resulting in minimal changes in the voltammetric response. Notably, the calculated IC_50_ value for ATA was an impressive 0.096 µM, highlighting the exceptional sensitivity of our platform for detecting and quantifying EcoRV inhibition.

**Figure 5:**
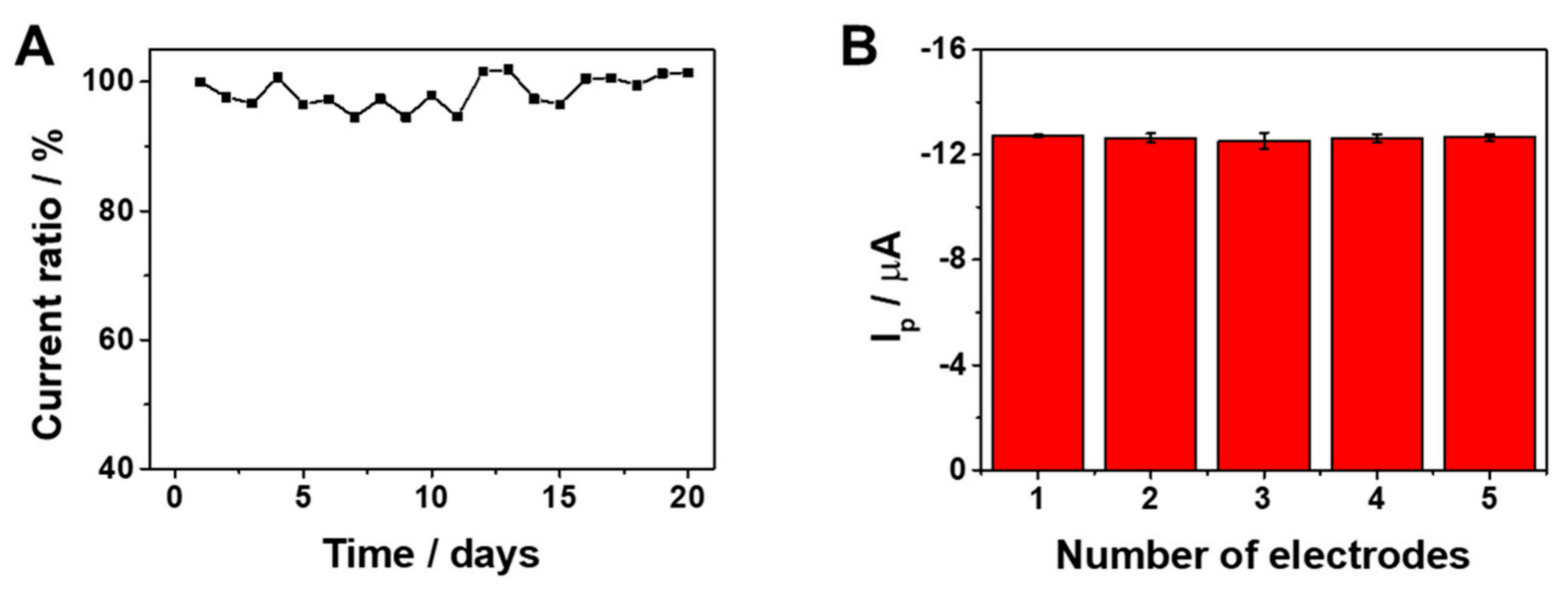
(**A**) Stability of the sensor in twenty days. (**B**) Reproducibility of the sensor for five different fabricated electrodes. The current was measured in triplicate (*n* = 3).

The reliability and practical application of the sensor depend on its reproducibility and stability. The proposed sensor demonstrated excellent stability, as illustrated in Figure 5A, where the voltammetric response was maintained at 101% over a span of 20 days. Figure 5B displays the I_p_ with minimal variation from five independently fabricated electrodes, highlighting the consistent performance achievable when using this fabrication method. 

## 4. Conclusions

This work presents a novel and versatile electrochemical platform for analyzing both EcoRV activity and inhibition, demonstrating significant advantages over current methods. The assay leverages readily prepared DNA substrates and simple electrochemical measurements to enable rapid and efficient analysis. Its remarkable sensitivity, with a detection limit of 9.5 × 10⁻^3^ U mL⁻^1^, and excellent selectivity through specific target sequence recognition, surpass previous approaches. Notably, the platform retains responsiveness even in the presence of a known inhibitor like ATA, making it well suited for drug discovery and clinical diagnostics. These combined strengths position this method as a promising tool with diverse applications. Further optimizations could expand its capabilities to target other enzymes or analyze complex biological samples. In conclusion, this study paves the way for advancements in enzyme activity and inhibition analysis, holding immense potential for research and diagnostic applications in various fields.

## Figures and Tables

**Table 1:** Comparison of previously reported methods for the determination of EcoRV activity.

Method	Detection Material	Linear Range	LOD	Reference
Fluorescence	EcoRV	0.1–500 U/mL	0.03 U/mL	[32]
	EcoRV	0.025–3.75 U/mL	0.0115 U/mL	[38]
FRET	EcoRV	25–500 U/mL	23.3 U/mL	[39]
Resonance light scattering (RLS)	EcoRV	−62.5 U/mL	0.016 U/mL	[2]
Electrochemistry	EcoRV	100–600 U/mL	-	[23]
	EcoRV	0.01–0.2 U/mL	0.0095 U/mL	This work

## Data Availability

Data are contained within the article and Supplementary Materials.

## Supplementary Materials

The following supporting information can be downloaded at: https://www.mdpi.com/article/10.3390/bios14020073/s1.
