# Supplementary material for: Electrochemical DNA Cleavage Sensing for EcoRV Activity and Inhibition with an ERGO Electrode
Source: Biosensors (Basel). 2024 Jan 30;14(2):73. doi: 10.3390/bios14020073 (PMC10886839; doi:10.3390/bios14020073)
Supplement: Supplementary file 1 [file biosensors-14-00073-s001.zip › biosensors-2804742-supplementary.pdf]

Supplementary Materials

# Electrochemical DNA Cleavage Sensing for EcoRV Activity and Inhibition with an ERGO Electrode

*Da Eun Oh[§], Hyun Beom Kim[§], Tae Hyun Kim**

Department of Chemistry, Soonchunhyang University, Asan 31538, Republic of Korea

[§]These authors contributed equally to this work.

*Correspondence should be addressed to T. H. Kim (thkim@sch.ac.kr)

**Table S1.** Source and recognition site of EcoRV.

| Enzyme | Source | Recognition Sequence | Cut |
|---|---|---|---|
| EcoRV | Escherichia coli | 5'GATATC<br>3'CTATAG | 5'---GAT ATC---3'<br>3'---CTA TAG---5' |

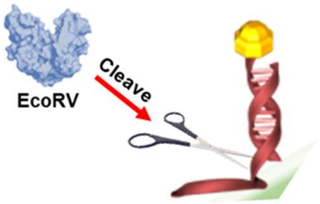


**5′-CTA GCT ATG TGC CGA ATT TCA AGG ACA GTT GTA TGG AT A TCA TAC T-3′**
**3′-ACC TA T AGT ATG A-5′**

**Table S2.** Contact angle measurements of water droplets on each electrode.

| | Bare GCE | ERGO-GCE | DNA/ERGO-GCE |
|---|---|---|---|
| Average | 76.44° | 79.19° | 53.25° |
| Standard Deviation (n=3) | 0.40 | 0.14 | 1.61 |
| | 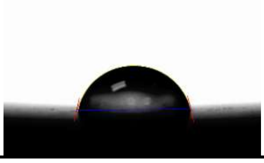 | 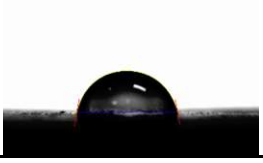 | 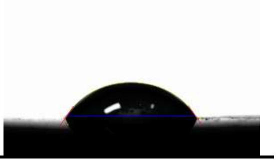 |
